# Metabolite profiling of four Tunisian *Eucalyptus* essential oils and assessment of their insecticidal and antifungal activities

**DOI:** 10.1016/j.heliyon.2023.e22713

**Published:** 2023-11-22

**Authors:** Sana Khedhri, Marwa Khammassi, Sonia BOUKHRIS. Bouhachem, Ylenia Pieracci, Yassine Mabrouk, Emine Seçer, Ismail Amri, Guido Flamini, Lamia Hamrouni

**Affiliations:** a Laboratory of Management and Valorization of Forest Resources, National Institute of Researches on Rural Engineering, Water and Forests, P.B. 10, 2080, Ariana, Tunisia; b Faculty of Science, Bizerte, Zarzouna 7021, Tunisia; c INRAT - National Institute of Agronomic Research of Tunisia, Laboratory of Plant Protection, Carthage University, Ariana, Tunisia; d Dipartimento di Farmacia, via Bonanno 6, University of Pisa, 56126 Pisa, Italy; e Laboratory of Biotechnology and Nuclear Technology, National Center of Nuclear Science and Technology, Sidi Thabet, B.P. 72, 2020, Ariana, Tunisia; f Nuclear Energy Research Institute, Istanbul Road 30 Km Saray Mah. Atom Cad. No: 27, 06983 Kahramankazan, Ankara, Turkey

**Keywords:** Essentials oils, 1,8-Cineole, *β*-eudesmol, Contact toxicity, antifungal activity

## Abstract

Aphids (*Aphidoidea*) and *Fusarium* spp. are widely recognized as destructive pests that cause significant damage to crops on a global scale. This study aimed to ascertain the chemical composition of essential oils (EOs) of four Tunisian *Eucalyptus* species and evaluate their toxicity against common aphids and phytopathogenic fungi.

The EOs were obtained *via* hydrodistillation and subsequently analyzed using GC-MS. The chemical composition analysis revealed the presence of five distinct chemical classes in the EOs: monoterpene hydrocarbons (3.8–16.7 %), oxygenated monoterpenes (5.5–86.0 %), sesquiterpene hydrocarbons (0.2–2.2 %), oxygenated sesquiterpenes (4.2–86.7 %), and non-terpene derivatives (0.1–14.1 %).Hierarchical clustering analysis (HCA) and principal component analysis (PCA) of the *Eucalyptus* leaf EOs highlighted significant differences among them, leading to the generation of distinct HCA clades representing at least twelve major components.

The statistical analysis clearly demonstrated a dose-response relationship, indicating the impact of the tested EOs on the growth of insects and fungal mycelium. The observed effects varied due to the variability in the chemical compositions of the EOs.

Notably, among the EOs tested, Eucalyptus lesoufii Maiden exhibited particularly potent effects against the targeted insect and fungal species. This research contributes to the ongoing exploration of natural alternatives to chemical pesticides, providing further insights for potential industrial applications. It underscores the versatility of these EOs and their potential as valuable candidates in strategies for pest and disease management.

## 1 Introduction

The agricultural sector plays a crucial role in meeting the ever-growing demand for food and agri-food products. However, it faces persistent challenges due to pests and diseases, significantly affecting agricultural productivity [1,2].

The historical reliance on chemical pesticides for pest control and increased food production has led to various issues, including pest resistance and adverse health effects [3]. Additionally, the use of agrochemicals negatively influences the environment, causing contamination of the atmosphere, soil, groundwater, and surface water through runoff, leaching, and spraying processes [4]. Furthermore, synthetic pesticides have been associated with various health complications in humans, ranging from mild sensitivities and respiratory difficulties to reproductive and neurotoxic disorders, and even chronic diseases [5,6].

Addressing these pressing concerns has evolved into a global imperative, with a primary emphasis on the adoption of integrated pest management strategies [7]. One promising avenue involves the exploration of plant species and their secondary metabolites, particularly EOs, as potential substitutes for synthetic chemicals. EOs are intricate blends of bioactive compounds, encompassing terpenes, phenols, and aldehydes, which collectively collaborate to combat both insect and fungal pathogens. Utilizing botanical antifungal and insecticidal agents offers numerous advantages over synthetic chemical insecticides and fungicides. These include reduced environmental impact, biodegradability, and a potentially diminished risk of fungal resistance development [8]. Consequently, there is a mounting interest in investigating the efficacy of botanical sources like EOs as sustainable and environmentally friendly alternatives for managing fungal diseases across various domains, such as agriculture, horticulture, and healthcare [9,10]. Due to their ecological compatibility and rapid degradation in the environment, EOs have emerged as attractive candidates for biologically-driven alternatives to traditional synthetic chemicals [11,12].

Among the families of plants known for their efficacy against pests, the Myrtaceae family, especially the genus *Eucalyptus*, has gained recognition for its biological and pharmaceutical properties. In Tunisian traditional medicine, *Eucalyptus* EOs are commonly used to treat respiratory disorders like bronchitis, sinusitis, and pharyngitis. Research has demonstrated the effectiveness of *Eucalyptus globulus* Labill. EOs against respiratory tract infections, including antibiotic-resistant strains [13]. Moreover, recent studies have highlighted the antibacterial and potential anti-biofilm activities of various *Eucalyptus* EOs [14,15].

Furthermore, several studies have reported the antifungal properties of EOs extracted from *Eucalyptus* species. Notably, EOs from *Eucalyptus camaldulensis* Dehnh. , *Eucalyptus citriodora* (Hook.) K.D.Hill*, Eucalyptus urophylla* S.T.Blake, and *Eucalyptus grandis* W.Hill ex Maiden, have shown effective inhibition of mycelial growth against various phytopathogenic fungi [16]. However, limited research has focused on the toxicity of *Eucalyptus* EOs against aphids, which are important pests and vectors of viruses affecting numerous crops in greenhouses and open fields [16].

The aims of this investigation were to examine the antifungal and insecticidal activities of four Tunisian *Eucalyptus* EOs. In particular, the study involved an analysis of the chemical composition of EOs derived from *Eucalyptus longicornis* F.Muell Maiden, *Eucalyptus obliqua* L'Hér, *Eucalyptus grifthsii* Maiden, and *Eucalyptus lesoufii* Maiden. Furthermore, the current study sought to evaluate the toxicity of these oils against *Aphis nerii* Boyer de Fonscolombe, *Aphis fabae* Scopoli, and *Planococcus citri* Risso. Additionally, their potential antifungal properties were assessed against various fungal strains, including *Fusarium lycopersici* Schltdl, *Fusarium redolens* Schltdl, and *Fusarium culmorum* Schltd.

## 2 Materials and methods

### 2.1 Plant materials

The plant material used in this study was collected during the spring season (April–May 2020), from the HINCHIR NAAM arboretum, which are located in the semi-arid region of Siliana-Tunisia. This arboretum are part of the National Institute of Researches on Rural Engineering, Water, and Forests.

For each of the selected *Eucalyptus* species, namely *Eucalyptus obliqua*, *E. lesoufii, E. grifthsii, and E. longicorni,* five leaf samples were gathered from more than five different trees. These samples were combined to ensure homogeneity.

Dr. Lamia Hamrouni identified the samples, and the voucher specimens (EO202, EL203, EG204 and ELO205 respectively) were deposited in the herbarium section of the Institute.

Subsequently, the representative homogenous samples of each species were placed in a greenhouse and allowed to dry in the shade for a period of 3–5 days until a constant weight was reached.

### 2.2 Essential oils extraction

EOs, were obtained by hydrodistillation of dried leaf samples (200 g for each species). The hydrodistillation process was carried out for 3h using a Clevenger apparatus, following the standard procedure outlined in the European Pharmacopoeia [17]. Extraction procedure was repeated three times to ensure thorough extraction. Obtained oils were collected, dried using anhydrous sodium sulfate, and stored in sealed glass brown vials in a refrigerator at 4 °C until further analysis and bioassay studies.

The yield of EOs was determined based on the dried weight of the initial sample (expressed as W/W %).

### 2.3 Gas chromatography and mass spectrometry analysis

Gas chromatography/Electron Ionization –Mass Spectrometry (GC/EI-MS) were performed

using an Agilent 7890 B gas chromatograph (Agilent Technologies Inc. Santa Clara, CA,

USA). Equipped with an Agilent HP-5MS capillary column (30 m × 0.25 mm; coating thickness.

0.25 μm) and an Agilent 5977B single quadrupole mass detector.

The analysis conditions were as follows: oven temperature programmed from 60 °C to.

240 °C at 3 °C/min; injector temperature 220 °C; transfer temperature 240 °C; carrier gas

helium at a flow rate of 1 mL/min. Injection of 1 μl of Eos diluted (5 %) in HPLC –grade *n*-hexane.

The acquisition parameters are specified as follows: full scan; scanning range: 35-300 m/z; sampling time: 1.0 second; threshold: 1 counter.

Components were identified based on comparing their retention times to those of pure

reference samples and comparing their linear retention indices (LRI) to the series of *n*-alkanes.

Mass spectra were compared to those listed in commercial libraries NIST 14 and Adams.

[18,19], and to homemade mass spectral libraries constructed using MS literature combined

with data obtained experimentally from pure substances.

### 2.4 Contact toxicity bioassay

*Aphis fabae, Aphis nerii,* and *Planococcus citri* insects were obtained from the Laboratory of Plant Protection at the National Institute of Agronomic Research in Tunisia.

All insects were reared under standard conditions, with a temperature range of 23–27 °C, relative humidity of 65 ± 5 %, and a light-dark photoperiod of 16:8 h. The selected individuals used for the bioassays were not gender-specific.

To conduct contact toxicity bioassays (tarsal, ventral, and lip contact), ten wingless individuals were carefully transferred using a fine brush into Petri dishes containing treated filter papers. The filter papers were placed on untreated fresh leaves, which served as a food source for the aphids.

Three different doses of 0.2, 0.4, and 0.6 mg/mL were tested for all oils. The control group consisted of a water solution containing 2 % Tween 20. Each treatment was replicated three times. Aphid mortality was observed 24 h after exposure to the EOs, and a dead aphid was defined as having no movement in its antennae or legs [20], as per the criteria established by Abbott in 1925 [21].

To calculate corrected mortality, the modified Abbott formula was used, which takes into account the mortality observed in the treated Petri dishes (Mo) and the natural mortality in the control group (Mt). The formula used was:

Mc = [(Mo - Mt)/(100 - Mt)] × 100.

To estimate the LD_50_ and LD_90_ values, PROBIT analysis was performed with 95 % confidence intervals for the lower and upper values, following the methodology outlined by Finney in 1971 [22].

### 2.5 Antifungal bioassay

Three fungal species, namely *Fusarium redolens*, *F. lycopercisii*, and *F. culmorum*, were used. These fungal strains were obtained from the Turkish Institute of Nuclear and Mining Energy Research. Antifungal evaluation was conducted through an *in vitro* contact bioassay that assessed the inhibition of hyphal growth.

To perform the bioassay, plates were prepared by dissolving the EOs in 1 ml of Tween 20 (0.1 % v/v) and adding it to 20 ml of Potato Dextrose Agar (PDA), a commonly used culture medium containing potato infusion and dextrose. The mixture was maintained at 50 °C. A 5 mm diameter mycelial disc, taken from the periphery of a 7day culture, was inoculated into the center of each PDA plate (90 mm diameter). The plates were then incubated in the dark at 24 °C for 7 days.

Four different doses of 2, 4, 6, and 10 mg/mL were tested for all tested oils. A PDA plate containing only Tween 20 (0.1 %) was used as a negative control. The percent radial growth inhibition relative to the control was used to assess the growth inhibition values calculated using the following equation:

Percent inhibition (%) = (C - T)/C * 100.Where:

C represents the mean hyphal elongation (mm) of the triplicate controls, and.

T represents the mean value of the three replicates of hyphal elongation (mm) of the plate treated with the EOs [23].

### 2.6 Statistical analysis

Data were analyzed with SPSS software, Student-Newman-Keuls SNK were used to test for variances between the means, and all *P*-values ≤0.05 were considered significantly different. LC_50_: Lethal concentrations were calculated using mortality rates obtained after 24 h in bioassay by PROBIT analysis, ANOVA test at *P*-values ≤0.05 was used to compare aphid mortality of tested EOs.

## 3 Result and discussion

### 3.1 Yields and chemical composition

The hydrodistillation of *Eucalyptus* leaves resulted in the production of yellow oils. Specifically, *E. obliqua*, *E. longicornis*, *E. griffthsii*, and *E. lesoufii* yielded oil percentages (W/W) of 1.23 ± 0.56 %, 1.78 ± 0.76 %, 1.62 ± 0.18 %, and 2.10 ± 0.6 %, respectively.

**Fig. 1:**
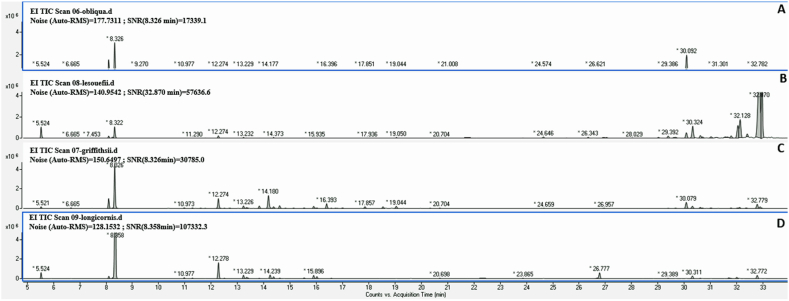
Chromatograms of *Eucalyptus obliqua* (A), *Eucalyptus lesouefii* (B), *Eucalyptus griffthsii* (C) and *Eucalyptus longicornis* (D).

The analysis of these oils using GC/MS (Fig. 1) identified a total of 64 components across the four *Eucalyptus* species, comprising 98.0 %–99.0 % of the total oils. These components fell into five chemical classes: monoterpene hydrocarbons (3.8–16.7 %), oxygenated monoterpenes (5.5–86.0 %), sesquiterpene hydrocarbons (0.2–2.2 %), oxygenated sesquiterpenes (4.2–86.7 %), and non-terpene derivatives (0.1–14.1 %).

The chemical composition analysis (Table 1-Fig. 4) revealed variations in the *Eucalyptus* EOs among different species. Notably, oxygenated monoterpenes were most abundant in *E. longicornis, E. obliqua,* and *E. griffthsii* EOs, ranging from 41.1 % to 86.0 %.

**Table 1:** Chemical composition of *Eucalyptus* EOs.

Peaks	Compounds	LRI_a_	LRI_b_	*Eucalyptus obliqua*	*Eucalyptus griffthsii*	*Eucalyptus Lesouefii*	*Eucalyptus longicornis*
Monoterpene hydrocarbons				16.7	8.2	3.8	3.8
1	α-pinene **(α-pin)**	941	939	3.9	1.0	2.7	2.3
2	β-pinene	982	979	1.2	0.4	0.1	–
3	α-phellandrene	1006	1002	–	–	0.1	–
4	*p*-cymene **(*p*-cym)**	1028	1024	10.4	6.5	0.6	1.1
5	limonene	1032	1029	1.0	0.3	0.3	0.4
6	γ-terpinene	1063	1017	0.2	–	–	–
Oxygenated monoterpenes				41.1	58.9	5.5	86.0
7	1,8-cineole **(1,8-cine)**	1034	1031	21.4	30.8	3.2	67.9
8	fenchol	1112	1116	0.2	0.4	0.1	0.3
9	*cis*-*p*-menth-2-en-1-ol	1123	1121	–	0.2	–	–
10	α-campholenal	1126	1126	–	0.2	0.1	–
11	*trans*-pinocarveol **(tr-pino)**	1141	1139	4.7	8.0	0.8	7.9
12	camphor	1144	1146	–	0.2	–	0.2
13	pinocarvone (**pinoc**)	1164	1164	1.2	2.0	0.2	1.8
14	borneol	1166	1169	–	0.9	0.1	0.7
15	*trans*-ocimenol	1169	1168.5	–	–	–	0.2
16	4-terpineol	1179	1177	0.8	1.9	0.1	0.2
17	*p*-mentha-1(7),8-dien-2-ol	1186	1189	0.4	–	0.1	1.7
18	α-terpineol	1191	1188	1.0	1.4	0.2	0.9
19	myrtenol	1193	1195	–	–	0.1	0.3
20	myrtenal **(myr)**	1164	1195	2.2	2.3	–	–
21	verbenone	1206	1205	0.3	0.3	–	–
22	*trans*-carveol	1220	1216	0.3	0.4	0.1	0.4
23	*cis*-carveol	1228	1229	–	–	–	1.8
24	*(Z)*-tagetone	1231	1159	–	0.4	–	1.2
25	cumin aldehyde (**cum alde**)	1241	1241	2.6	3.6	–	–
26	carvone	1244	1243	–	0.2		0.2
27	carvotanacetone	1247	1247	–	0.2	–	–
28	piperitone	1254	1252	0.3	0.5	–	–
29	phellandral	1275	1259	1.6	1.5	–	–
30	citronellyl formate	1276	1273	–	–	0.1	–
*31*	*p*-cymen-7-ol	1290	1290	1.4	1.5	–	–
32	carvacrol (carv)	1298	1299	2.0	1.7	0.2	
33	2-acetoxy-1,8-cineole	1345	1344	0.3	0.3	0.1	0.3
34	α-terpinyl acetate	1352	1349	0.4	–	–	–
Sesquiterpene hydrocarbons				0.9	0.7	2.2	0.2
35	β-caryophyllene	1419	1408	–	–	0.1	0.2
36	aromadendrene	1440	1441	0.2	0.2	0.4	–
37	alloaromadendrene	1462	1460	–	–	0.1	–
*38*	*cis*-muurola-4(14),5-diene	1463	1466	–	–	–	–
39	germacrene D	1482	1481	–	–	0.3	–
40	α-vetispirene	1488	1490	0.4			–
41	viridiflorene	1494	1496	–	–	0.2	–
42	bicyclogermacrene	1496	1500		0.3	0.2	–
43	δ-cadinene	1524	1523	–	–	0.1	–
44	germacrene B	1557	1561	0.3	0.2	0.8	–
Oxygenated sesquiterpenes				32.2	16.1	86.7	4.2
45	β-dihydroagarofuran	1497	1520	–	–	0.3	–
46	elemol	1550	1549	–	–	0.3	–
47	ledol	1566	1602	–	0.3	0.4	–
48	spathulenol**(spath)**	1576	1578	17.8	5.6	2.0	–
49	globulol **(glob)**	1583	1590	4.3	2.2	4.5	1.8
50	viridiflorol	1591	1592	0.8	–	1.0	0.3
51	rosifoliol	1601	1600	–	0.5	0.6	–
52	10-*epi*-α-eudesmol (**10-*epi*-α-eud**)	1620	1623	0.5	0.4	5.1	0.2
53	γ-eudesmol **(γ-eu)**	1631	1632	0.3	1.0	7.0	–
54	isospathulenol	1640	1639	1.9	0.5	–	–
55	β-eudesmol**(β-eud)**	1650	1650	5.2	4.0	44.9	1.9
56	α-eudesmol**(α –eud)**	1651	1653	1.1	1.6	20.2	–
Non-terpene derivatives				7.4	14.1	0.1	4.8
57	isopentyl isovalerate	1104	1103	0.5	0.5	–	0.5
58	cryptone	1185	1185	4.4	11.6	–	–
59	*p*-cumenol	1229	1227	1.5	1.9	–	–
60	isoamyl benzoate	1439	1435	1.0	0.1	0.1	–
61	isoamyl phenyl acetate	1489	1477	–	–	–	0.4
62	β-phenylethyl isovalerate	1490	1491	–	–	–	3.3
63	leptospermone	1619	1630	–	–	–	0.6
Total identified%				98.3	98.0	98.3	99.0

LRI_a:_ calculated retention index,LRI_b_: Literature retention Index, -: not detected.

*E. longicornis* EOs had the lowest number of components, totaling 28. The predominant compounds were oxygenated monoterpenes, with a significant presence of 1,8-cineole (constituting 67.9 %), *trans*-pinocarveol (7.9 %), and pinocarvone (1.8 %). Oxygenated sesquiterpenes made up 4.2 % of the composition, with *β*-eudesmol (1.9 %) and globulol (1.8 %) being the major constituents in this category. Monoterpene hydrocarbons, including *α*-pinene (2.3 %) and *p*-cymene (1.1 %), accounted for 3.8 % of the total oil.

Concerning the *E. obliqua* EO, a total of 38 components were identified. These components were divided into oxygenated monoterpene and oxygenated sesquiterpene fractions, constituting 41.1 % and 32.2 % of the EO, respectively. Among the noteworthy oxygenated monoterpenes were 1,8-cineole (21.4 %), *trans*-pinocarvone (4.3 %), cumin aldehyde (2.6 %), and myrtenal (2.2 %). As for the oxygenated sesquiterpenes, the major constituents included spathulenol (17.8 %), *β*-eudesmol (5.2 %), and globulol (4.8 %). Additionally, there was a presence of monoterpene hydrocarbons, accounting for 16.7 % of the oil, with *p*-cymene (10.4 %) and α-pinene (3.9 %) as the primary compounds in this category.

EO derived from *E. griffthsii* exhibited the highest number of components, totaling 42. The most prominent phytochemical group within this EO was the oxygenated monoterpenes, comprising 58.9 % of the composition. Noteworthy constituents in this category included 1,8-cineole (constituting 30.8 % of the EO), *trans*-pinocarveol (8 %), and cumin aldehyde (3.6 %). Oxygenated sesquiterpenes accounted for 16.1 % of the composition, with major representatives being spathulenol (5.6 %), globulol (2.2 %), and eudesmol isomers, including *β*-eudesmol (4 %), *α*-eudesmol (1.6 %), and *γ*-eudesmol (1 %). Additionally, non-terpene derivatives contributed 14.1 % to the oil, with cryptone (11.6 %) as the predominant compound in this category. Monoterpene hydrocarbons made up 8.2 % of the total oil, with *p*-cymene (6.4 %) and *α*-pinene (1 %) being notable constituents within this group.

The class of sesquiterpene hydrocarbons exhibited the lowest presence, comprising only 0.2 %, 0.7 %, and 0.9 % in *E. longicornis*, *E. griffthsii*, and *E. obliqua*, respectively.

In the case of non-terpene derivatives, they were notably present in *E. obliqua* EO, accounting for 7.4 % of its composition, with constituents like cryptone (4.4 %) and isoamyl benzoate (1 %).

Conversely, the EO from *E. lesoufii* was distinguished by a notably high concentration of oxygenated sesquiterpenes, accounting for 86.7 % of the composition. Among these, the predominant compounds were the eudesmol isomers: *β*-eudesmol (constituting 44.9 % of the EO), *α*-eudesmol (20.2 %), *γ*-eudesmol (7 %), 10-*epi-α*-eudesmol (5.1 %), and globulol (4.5 %). In contrast, the proportion of oxygenated monoterpenes was relatively low, at less than 5.5 %, with 1,8-cineole representing 3.2 %. Additionally, monoterpene hydrocarbons accounted for 3.8 % of the total oil, with *α*-pinene making up 2.7 % of this category. Sesquiterpene hydrocarbons (2.2 %) were represented by germacrene B (0.8 %).

As per existing literature, there have been limited studies focusing on these particular species. Notably, it's worth mentioning that the chemical composition of *E. obliqua* EO extracted from Australian trees displayed variations compared to our findings. Specifically, previous research reported relatively elevated concentrations of certain compounds, including *p*-cymene (20 %), bicyclogermacrene (20 %), piperitone (15 %), *trans*-menth-2-en-1-ol (16 %), spathulenol (7 %), and *β*-phellandrene (7 %) [24].

Moreover, the EO from Tunisian *E. longicornis* exhibited similar major components with slight variations in their average concentrations, notably in the case of *α*-pinene and 1,8-cineole [25]. Additionally, the chemical composition of *E. lesoufii* EO reported by Elaissi et al. [26] differed from our findings, showing a significant abundance of the oxygenated monoterpenes fraction, particularly 1,8-cineole (38 %), and monoterpene hydrocarbons, primarily represented by *α*-pinene (12.8 %) and *β*-pinene (10.9 %). These disparities may be attributed to a variety of factors.

In fact, the composition of EOs not only varies among distinct species of aromatic plants but is also subject to variations due to the presence of different chemotypes and the influence of pedoclimatic conditions within each plant species [27,28].

**Fig. 2:**
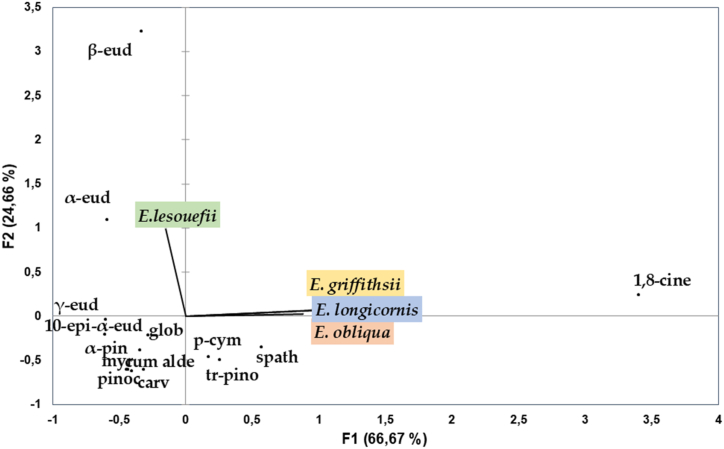
Principal component analysis (PCA) of 15 compounds for leaves EOs of *Eucalyptus* species.

**Fig. 3:**
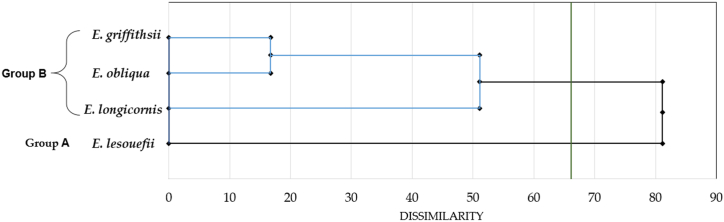
Dendrogram obtained by cluster analysis based on the Euclidean distances between groups of the four leaves EOs of Tunisian *Eucalyptus* species.

For PCA and HCA, fifteen major compounds with average concentrations exceeding 2 % were selected (Figs. 2 and 3).

**Fig. 4:**
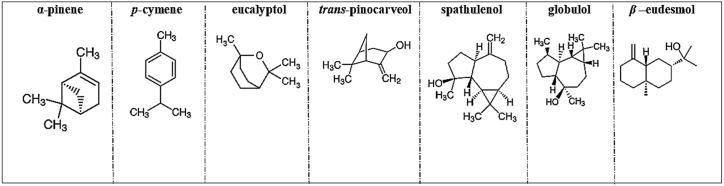
Chemical structures of some compounds isolated from Tunisian *Eucalyptus* EOs.

The PCA applied to *Eucalyptus* EOs resulted in the extraction of two principal components, which were represented by the horizontal and vertical axes. These components explained 66.67 % and 24.66 % of the total variance, respectively.

The HCA, based on Euclidean distances between species groups, revealed the presence of two distinct groups, denoted as Group A and Group B, with a dissimilarity greater than 65.

Upon closer examination of Group B, it was subdivided into two subgroups, specifically B1 and B2, with a dissimilarity exceeding 50. Subsequently, the B2 subgroup was further divided into two additional groups, characterized by a dissimilarity greater than 15. It's noteworthy that even within the same group, there were significant variations in the chemical composition of the EOs.

Group A, which consisted solely of *E. lesoufii*, displayed unique characteristics in both PCA and HCA analyses. These characteristics included elevated levels of specific compounds such as *β*-eudesmol (44.9 %), *α*-eudesmol (20.9 %), *γ*-eudesmol (7 %), 10*-epi-α*-eudesmol (5.1 %), and a relatively lower percentage of eucalyptol (3.2 %) in the composition of the EOs within this group.

In contrast, the primary distinguishing factor among the three species within Group B was associated with *E. longicornis*, which exhibited significantly higher levels of eucalyptol (67.9 %) compared to the other two species (21.4 % and 30.8 %). Within the B2 subgroup, which included *E. obliqua* and *E. griffthsii*, the dissimilarity between these species was greater than 5. Notably, these species presented varying proportions of compounds such as *p*-cymene (ranging from 10.4 % to 6.5 %), *trans*-pinocarveol (ranging from 4.7 % to 8 %), and spathulenol (ranging from 17.8 % to 5.6 %) in their respective EOs.

The statistical analysis of the chosen components in the EOs unveiled considerable variability. Both the HCA and PCA conducted on the *Eucalyptus* leaf EOs emphasized substantial distinctions between the groups. The HCA analysis resulted in the identification of a minimum of twelve major components, each represented by distinct branches. This analysis revealed that each group of species possessed a unique chemotype.

### *3.2* *In vitro* contact bioassays

Tables 2 and 3 display the outcomes of insecticidal bioassays carried out using *Eucalyptus* EOs against *Aphis fabae*, *A. nerii*, and *Planococcus citri*. In laboratory tests, it was observed that the corrected mortality rate progressively increased with higher concentrations of EOs applied, particularly after 24 h exposure period.

**Table 2:** Contact activity of essential oils derived from *Eucalyptus* leaves against *Aphis fabae, A. nerii And Planococcus citri* adults.

Insect species	Doses *(*mg/mL)	*Eucalyptus lesouefii*	*Eucalyptus griffthsii*	*Eucalyptus obliqua*	*Eucalyptus longicornis*
*Aphis fabae*	0.2	46.66 ± 6.29a	39.44 ± 1.09a	37.22 **±** 2.54a	29.55 **±** 1.03a
	0.4	50.83 ± 1.44b	51.11 ± 1.92b	41.38 **±** 4.28b	37.22 **±** 2.43b
	0.6	75.00 ± 2.50c	76.00 ± 1.92c	66.38 **±** 6.02c	61.91 **±** 2.43c
*Aphis nerii*	0.2	28.93 ± 1.77a	24.44 ± 1.92a	28.88 **±** 5.09a	27.97 **±** 1.92a
	0.4	39.80 ± 1.09b	31.11 ± 1.92b	30.99 **±** 4.04b	35.53 **±** 6.67b
	0.6	65.95 ± 2.09c	52.22 ± 5.09c	36.55 **±** 3.34c	59.92 **±** 0.00c
*Planococcus citri*	0.2	73.60 ± 2.5a	46.66 ± 3.33a	64.16 **±** 1.44a	68.00 **±** 1.75a
	0.4	80.64 ± 0.46b	62.22 ± 1.09b	72.83 **±** 1.60b	76.84 **±** 1.63b
	0.6	95.95 ± 1.44c	87.33 ± 2.77c	94.00 **±** 1.29c	93.33 **±** 0.57c

Values are means ± standard errors means (n = 3); means followed by the same letter in the same column are not significantly different by the Student_Newman_Keuls test (*P* ≤ 0.05).

**Table 3:** Lethal concentrations of essential oils derived from *Eucalyptus* leaves against *Aphis fabae, A. nerii And Planococcus citri* after 24h exposure.

Insect species	Essentials oils	LC_50_ (mg/mL)	LC_90_ (mg/mL)	CI (%)	χ2	*P*
*Aphis fabae*	*E. lesouefii*	0.264	2.124	0.677–2.154	5.570	0.018
	*E. griffthsii*	0.304	1.447	1.144–2.839	4.087	0.043
	*E. obliqua*	0.390	3.049	0.698–2.171	5.33	0.021
	*E. Longicornis*	0.472	2.782	0.914–2.414	4.028	0.045
*Aphis nerii*	*E. lesouefii*	0.429	1.988	1.169–2.679	3.886	0.049
	*E. griffthsii*	0.585	3.393	0.913–2.445	4.147	0.042
	*E. obliqua*	0.938	23.195	0.171–1.669	2.140	0.143
	*E. Longicornis*	0.504	2.984	0.901–2.414	3.878	0.049
*Planococcus Citri*	*E. lesoufii*	0.053	0.307	0.608–2.735	3.877	0.05
	*E. griffthsii*	0.845	0.235	1.528–3.086	5.053	0.025
	*E. obliqua*	0.146	0.631	1.184–2.855	7.338	0.007
	*E. Longicornis*	0.210	0. 608	0.979–2.672	4.194	0.022

*LC* – lethal concentration causing 50 and 90 % mortality; *CI* – confidence interval; χ2 – chi-squared value for the lethal concentrations and fiducial limits based on a log scale with significance level at *P* ≤ 0.05.

Each of the EOs exhibited notable toxicity against the tested pests in the bioassay, although the LC_50_ values varied depending on the insect species and the particular EOs employed. These differences in toxicity can be attributed to the unique responses of the pests to the specific compounds present in the EOs.

The LC_50_ and LC_90_ values clearly indicated that *E. lesoufii* EO displayed the highest level of toxicity against the tested insects. Specifically, its LC_50_ values were measured at 0.429 mg/mL for *A. nerii*, 0.264 mg/mL for *A. fabae*, and an impressively low 0.053 mg/mL for *P. citri.*

In terms of the corrected mortality rate, it was observed that *P. citri* exhibited a higher susceptibility to the EOs compared to *A. nerii* and *A. fabae*. This difference in susceptibility could be attributed to variations in factors such as size, sensitivity to toxic vapors, and detoxification rates among these insect species.

It's worth noting that existing literature supports the notion that numerous plant-derived EOs possess insecticidal properties [29,30]. While *Eucalyptus* EOs have been reported to be toxic to coleopteran pests [31,32] and lepidopteran pests [33]. Yet, there is relatively limited documentation regarding their aphicidal properties.

*Eucalyptus citriodora* has exhibited noteworthy effects against *Myzus persicae* Sulzer, a pest that impacts citrus trees [34]. In a recent study conducted by Pathak et al. [35], both *Eucalyptus globulus* and its constituent, 1,8-cineole, demonstrated high efficacy against *A. fabae*, resulting in a mortality rate of 81.08 ± 6.2 % after 24 h. Similarly, Russo et al. [36] reported a 100 % mortality rate after 24 h for *Eucalyptus globulus* EO when used against *A. nerii*.

Similarly, Ebrahimi et al. [37] recently conducted an investigation into the notable insecticidal properties of *Eucalyptus camaldulensis* EO, characterized by their substantial concentrations of eucalyptol and *p*-cymene, in combating *Aphis gossypii* Glover. This study reaffirmed the potent insecticidal efficacy inherent in *Eucalyptus* EOs.

Considering their origin as lipophilic secondary metabolites from plants, EOs are primarily composed of terpenoids. Researchers have ascribed the insecticidal prowess of these EOs to the predominant compounds they contain, each possessing distinct physical and chemical attributes. For instance, eucalyptol has been documented as exhibiting broad insecticidal activity against various pests, including stored grain beetles [38,39] human lice [40,41] and German cockroaches [42]. Similarly, 1,8-cineole has been observed to induce hyperactivity in *Triatoma infestans* Klug [43]

*β*-eudesmol, isolated from *Atractylodes lancea* Thunb, has demonstrated effective repellent and contact activities against *Tribolium castaneum* Herbst adults [44]and has exhibited toxicity against a range of pests such as fruit flies, *Culex pipiens* Linnaeus [45], *Liposcelis bostrychophila* Badonnel [46], and red flour beetles [47]. The toxicological effects can be elucidated through various biochemical and physiological processes [48].

Furthermore, a substantial body of research has demonstrated the neurotoxic effects of EOs, particularly in insects, where they induce paralysis, ultimately resulting in mortality. This distinctive property has prompted investigations into the potential of EO components as bioinsecticides [49]. While a significant portion of research has centered on the inhibition of acetylcholinesterase (AChE) as a primary mechanism of EO action, it is noteworthy that EOs typically exhibit relatively modest AChE inhibitory activity [50,51]. An alternative hypothesized mechanism of EO action involves the positive allosteric modulation of GABA receptors (GABArs). Extensive scientific literature substantiates the enhancement of the GABAergic response in mammalian receptors induced by EOs [52,53].

However, the potential synergistic effects of complex or binary mixtures of monoterpenes on aphid mortality remain incompletely understood [54], [55]. This is due to the influence of terpenes and their derivatives on physiological responses through the octopaminergic system. Accumulating evidence suggests that EOs have the capability to elevate levels of both cyclic adenosine monophosphate (cAMP) and calcium within nerve cells. Moreover, specific components found in EOs can compete with octopamine for receptor binding [56,57].

Electrophysiological investigations conducted on *Periplaneta americana* Linnaeus have identified similarities in the actions of EO components and octopamine [58,59].

### *3.3* *In vitro* antifungal activity

Table 4 provides a summary of the antifungal activity, which was evaluated by measuring the diameter of fungal mycelium growth over a 7days period. The results clearly indicate that *Eucalyptus* EOs effectively reduced the growth of mycelia in all tested fungal strains when compared to the control.

**Table 4:** Antifungal activity of four *Eucalyptus* EOs.

Fungi strain	Doses mg/mL	*Eucalyptus griffithsii*	*Eucalyptus longicornis*	*Eucalyptus lesouefii*	*Eucalyptus obliqua*
*Fusarium culmorum*	4	53.89 ± 1.65a	69.92 ± 1.14a	75.39 ± 1.029a	53,51 ± 0,987a
	6	62.48 ± 3.33b	72.65 ± 0.78b	76.94 ± 0.829b	54,295 ± 0,308b
	8	75.56 ± 1.26c	76.07 ± 0.679c	86.65 ± 0.679c	56,078 ± 0,679c
	10	87.3 ± 0.64d	83.2 ± 0.74d	94.92 ± 0.695d	60,54 ± 0,59d
*Fusarium lycopersici*	4	43.87 ± 1.44a	50.00 ± 0.00a	52.91 ± 2.60a	50.00 ± 0.00a
	6	67.08 ± 0.721b	58.33 ± 0.72b	63.91 ± 1.01b	53.00 ± 0.72b
	8	75.87 ± 1.84c	66.667 ± 1.44c	73.33 ± 0.721c	59.58 ± 0.721c
	10	76.95 ± 0.93c	79.16 ± 0.72d	79.25 ± 0.661d	67.91 ± 1.44c
*Fusarium redolens*	4	51.25 ± 1.25a	73.33 ± 0.72a	71.66 ± 1.90a	62.53 ± 0.00a
	6	53.16 ± 1.445	75.75 ± 0.66b	77.91 ± 1.44b	68.75 ± 0.0b
	8	67.083 ± 1.443	78.085 ± 0.62c	82.083 ± 0.721c	70.38 ± 0.721c
	10	78.33 ± 0.72	85.00 ± 0.00d	86.25 ± 1.25d	75.00 ± 0.00d

Values are means ± standard errors means (n = 3); means followed by the same letter in the same column are not significantly different by the Student_Newman_Keuls test (*P* ≤ 0.05).

Statistical analysis reveals a pronounced dose-response effect, indicating a positive correlation between increasing concentrations of EOs and the inhibition percentage of mycelial growth.

Of particular significance is the effectiveness of tested EOs against *F. culmorum*, a widespread pathogenic species that impacts wheat and barley crops. At the highest concentration (10 mg/mL), the inhibitory effect of EOs consistently remained above 60.54 ± 0.59 % for *E. obliqua*, while *E. longicornis* and *E. griffthsii* achieved even higher inhibitory rates of 83.2 ± 0.74 % and 87.30 ± 0.64 %, respectively. *E. lesoufii* displayed nearly complete inhibition, reaching an impressive 94.92 ± 0.695 %.

Regarding *F. redolens*, the inhibitory percentage at a concentration of 10 mg/mL ranged from 75.00 ± 0.00 % for *E. obliqua* EO to 86.25 ± 1.25 % for *E. lesoufii* EO.

For *F. lycopersici*, the highest inhibitory percentage was observed at 10 mg/mL, with *E. lesoufii* EO showing an inhibition rate of 79.25 ± 0.6 %, while the lowest value was recorded with *E. obliqua* EO at 67.91 ± 1.44 %.

Among the various *Eucalyptus* EOs, *E. lesoufii* demonstrated the most potent activity against the tested fungal species. This heightened efficacy can be attributed to its elevated sesquiterpenes content, with *β*-eudesmol potentially being one of the primary compounds responsible for its inhibitory properties.

Numerous prior studies have consistently reported the antifungal activities of various EOs [60,61], highlighting their effectiveness in combating fungal infections. Furthermore, *β*-eudesmol, a prominent compound found in these EOs, has gained recognition for its diverse biological effects, including hypotensive, diuretic, and antimicrobial properties [62,63].

In a study conducted by Su and Ho [64], it was found that *Phoebe formosana* Hayata EOs exhibited potent antifungal activity against a wide range of fungal strains, with *β*-eudesmol identified as the active compound.

This discovery aligns with similar findings reported by Costa et al. [65], who observed significant antimicrobial activity, primarily attributed to *β*-eudesmol (51.60 %), in the EO obtained from *Guatteria friesiana* (W. A. Rodrigues) Erkens & Maas against 11 different microorganisms. The antimicrobial activity associated with *Eucalyptus* EOs is generally attributed to their abundant presence of oxygenated monoterpenes and sesquiterpenes [66].

Moreover, a prior study conducted by Amri et al. [67] reported the antifungal properties of EOs derived from *Eucalyptus citriodora*, *Eucalyptus sideroxylon* A.Cunn.exWoolls, and *Eucalyptus falcata* Turcz. against seven species of *Fusarium* spp, corroborating the findings of the current study. Numerous studies have consistently highlighted the potential of *Eucalyptus* EOs as highly effective antifungal agents.

For example, Gakuubi et al. [68] demonstrated the fungicidal properties of *Eucalyptus camaldulensis* EOs in managing *Fusarium* spp. Similarly, Tomazoni et al. [69] unveiled the fungicidal action of *Eucalyptus staigeriana* F.Muell. EO against phytopathogens such as *Alternaria solani* Sorauer and *Stemphylium solani* G.F.Weber.

Additionally, Lopez-Meneses et al. [70] reported the antifungal effect of *Eucalyptus globulus* EO against *Fusarium moniliforme* (Sacc.) Nirenberg and *Aspergillus parasiticus* Speare.

In the realm of fungal pathogen control, EOs have garnered considerable attention in the scientific literature due to their intricate and versatile mechanisms of action. Specifically, EOs are recognized for their capacity to disrupt fungal cell walls by establishing a membrane potential, subsequently disrupting ATP assembly, and ultimately resulting in damage to the fungal cell wall. Moreover, these oils possess the remarkable ability to disrupt both the mitochondrial membrane and the electron transport system (ETS) pathway within fungal cells [71].

This multifaceted impact on fungal physiology is comprehensively elucidated in the study conducted by Freiesleben et al. [72], revealing that the antifungal agents present in EOs target various aspects of fungal biology. These encompass not only the disruption of membrane structures but also the inhibition of nuclear materials and interference with protein synthesis. Notably, these compounds exhibit a remarkable capability to permeate fungal cells, interacting with intracellular sites [73]. Furthermore, akin to other plant-derived compounds, EOs demonstrate the potential to effectively hinder microbial growth and prevent the formation of biofilms through specific mechanisms. This comprehensive approach to fungal control, orchestrated by EOs through a synergy of compounds acting on diverse targets with varying mechanisms [74,75], imparts a significant advantage by reducing the likelihood of phytopathogens developing resistance to these natural agents [76].

## 4 Conclusion

Our investigation has unveiled the auspicious prospects associated with Tunisian *Eucalyptus* EOs as environmentally sustainable substitutes for chemical pesticides within the realm of agriculture. Notably, *E. lesoufii* has manifested exceptional bioactivity against a diverse spectrum of agricultural pests and pathogens, positioning it as a good contender for the future of sustainable pest control strategies.

The embrace of *Eucalyptus* EOs, grounded in their natural origins, signifies a pivotal stride towards the embodiment of eco-conscious agricultural practices. By reducing the reliance on synthetic chemical agents, we can proactively attenuate the pernicious repercussions these substances impose on ecosystems and their biodiversity.

Nonetheless, the realization of the full potential of *Eucalyptus* EOs necessitates a continued trajectory of rigorous investigation. Comprehensive assessments pertaining to their industrial applicability, efficaciousness, and long-term ecological ramifications must be earnestly pursued.

Additionally, the complexities surrounding the interactions between these natural compounds and the environment call for further elucidation and analysis.

## Data availability

Sharing research data helps other researchers evaluate your findings, build on your work and to increase trust in your article. We encourage all our authors to make as much of their data publicly available as reasonably possible. Please note that your response to the following questions regarding the public data availability and the reasons for potentially not making data available will be available alongside your article upon publications.

- Has data associated with your study been deposited into a publicity available repository?
- Data will be made available on request

## CRediT authorship contribution statement

**Sana Khedhri:** Data curation, Formal analysis, Writing - original draft. **Marwa Khammassi:** Data curation, Formal analysis, Software. **Sonia BOUKHRIS. Bouhachem:** Methodology, Resources, Software, Supervision. **Ylenia Pieracci:** Data curation, Formal analysis, Software, Writing - review & editing. **Yassine Mabrouk:** Methodology, Resources, Visualization. **Emine Seçer:** Resources, Writing - review & editing. **Ismail Amri:** Conceptualization, Methodology, Resources, Validation, Visualization, Writing - review & editing. **Guido Flamini:** Conceptualization, Methodology, Visualization, Writing - review & editing. **Lamia Hamrouni:** Conceptualization, Methodology, Resources, Validation, Visualization, Writing - review & editing.

## Declaration of competing interest

As the authors of this manuscript, we affirm that there are no conflicts of interest that could potentially bias the impartiality of the research presented herein. We further confirm that this research received no specific grants from funding agencies in the public, commercial, or not-for-profit sectors.

Moreover, we acknowledge that we have thoroughly read and understood Heliyon's policies on ethical publishing and hereby affirm that this manuscript adheres to those policies.
